# The effect of livestock density on *Trypanosoma brucei gambiense* and *T. b. rhodesiense*: A causal inference-based approach

**DOI:** 10.1371/journal.pntd.0010155

**Published:** 2022-08-29

**Authors:** Julianne Meisner, Agapitus Kato, Marshal Msanyama Lemerani, Erick Mwamba Miaka, Acaga Ismail Taban, Jonathan Wakefield, Ali Rowhani-Rahbar, David M. Pigott, Jonathan D. Mayer, Peter M. Rabinowitz

**Affiliations:** 1 Center for One Health Research, Department of Global Health, University of Washington, Seattle, Washington, United States of America; 2 Department of Epidemiology, University of Washington, Seattle, Washington, United States of America; 3 Uganda Virus Research Institute, Entebbe, Uganda; 4 Ministry of Health, Lilongwe, Malawi; 5 Programme National de Lutte contre la Trypanosomiase Humaine Africaine, Kinshasa, Democratic Republic of the Congo; 6 IntraHealth International, Juba, South Sudan; 7 Department of Biostatistics, University of Washington, Seattle, Washington, United States of America; 8 Department of Statistics, University of Washington, Seattle, Washington, United States of America; 9 Department of Health Metrics Sciences, University of Washington, Seattle, Washington, United States of America; 10 Department of Global Health, University of Washington, Seattle, Washington, United States of America; Institute of Tropical Medicine, BELGIUM

## Abstract

Domestic and wild animals are important reservoirs of the *rhodesiense* form of human African trypanosomiasis (rHAT), however quantification of this effect offers utility for deploying non-medical control activities, and anticipating their success when wildlife are excluded. Further, the uncertain role of animal reservoirs—particularly pigs—threatens elimination of transmission (EOT) targets set for the *gambiense* form (gHAT). Using a new time series of high-resolution cattle and pig density maps, HAT surveillance data collated by the WHO Atlas of HAT, and methods drawn from causal inference and spatial epidemiology, we conducted a retrospective ecological cohort study in Uganda, Malawi, Democratic Republic of the Congo (DRC) and South Sudan to estimate the effect of cattle and pig density on HAT risk.

For rHAT, we found a positive effect for cattle (RR 1.61, 95% CI 0.90, 2.99) and pigs (RR 2.07, 95% CI 1.15, 2.75) in Uganda, and a negative effect for cattle (RR 0.88, 95% CI 0.71, 1.10) and pigs (RR 0.42, 95% CI 0.23, 0.67) in Malawi. For gHAT we found a negative effect for cattle in Uganda (RR 0.88, 95% CI 0.50, 1.77) and South Sudan (RR 0.63, 95% CI 0.54, 0.77) but a positive effect in DRC (1.17, 95% CI 1.04, 1.32). For pigs, we found a positive gHAT effect in both Uganda (RR 2.02, 95% CI 0.87, 3.94) and DRC (RR 1.23, 95% CI 1.10, 1.37), and a negative association in South Sudan (RR 0.66, 95% CI 0.50, 0.98). These effects did not reach significance for the cattle-rHAT effect in Uganda or Malawi, or the cattle-gHAT and pig-gHAT effects in Uganda.

While ecological bias may drive the findings in South Sudan, estimated E-values and simulation studies suggest unmeasured confounding and underreporting are unlikely to explain our findings in Malawi, Uganda, and DRC. Our results suggest cattle and pigs may be important reservoirs of rHAT in Uganda but not Malawi, and that pigs—and possibly cattle—may be gHAT reservoirs.

## Author summary

Domestic animals, including cattle and pigs, are known to be important for the transmission of the *rhodesiense* form of human African trypanosomiasis (rHAT), however the relative importance of these reservoirs compared to wild animals is uncertain and likely focus-specific. For the *gambiense* form (gHAT) transmission is predominantly human-to-human, however pigs are thought to be a possible reservoir. In this study we used pre-existing data on livestock density and HAT risk to estimate the strength of the effect of cattle and pig density on rHAT risk in Uganda and Malawi, and gHAT risk in Uganda, Democratic Republic of the Congo (DRC), and South Sudan. We found evidence that cattle and pigs increase the risk of rHAT in Uganda but not Malawi, that pigs increase the risk of gHAT in Uganda and DRC, and that cattle increase the risk of gHAT in DRC alone. These results indicate that control of both forms of HAT should include domestic animals in a One Health framework, however control of rHAT in Malawi is unlikely to be achieved if measures exclude wild animals.

## Introduction

In recent decades, remarkable progress in the control of human African trypanosomiasis (HAT)—a disease caused by a bloodborne protozoal parasite transmitted by the tsetse (*Glossina* species)—has led the WHO to set targets for elimination as a public health problem (EPHP) by 2020, and elimination of transmission (EOT) by 2030. Global EPHP targets were met in 2018, however most endemic countries are not yet eligible for national EPHP validation, and there are significant challenges to achieving EOT goals [1].

Two forms of HAT, which are geographically- and epidemiologically-distinct, exist: the chronic form, caused by *Trypanosoma brucei gambiense* (gHAT) and endemic in western and central Africa, and the acute form, caused by *T. b. rhodesiense* (rHAT) and endemic in eastern and southern Africa. Both rHAT and gHAT present in two stages. The initial hemolymphatic stage, characterized by non-specific signs, is followed by the meningoencephalitic stage, characterized by central nervous system signs and eventual death in the absence of treatment, occurring within several weeks for rHAT and 18 months or longer for gHAT [2, 3].

Cattle are known to be an important reservoir of rHAT [4–8], and while pigs have historically been considered less important reservoirs due to their shorter lifespan, a survey conducted in Tanzania found 4.8% of domestic pigs harbored *T. b. rhodesiense* [9]. Numerous studies have also documented wildlife species to be competent rHAT reservoirs, and proximity to protected areas is a known risk factor for rHAT infection [10]. The zoonotic nature of rHAT complicates its control, leading to its exclusion from EOT goals [11] and significantly lower investment in rHAT surveillance and control compared with gHAT, raising concerns that rHAT will emerge as a major public health problem once gHAT EOT is achieved and donor attention moves away from HAT.

The omission of rHAT from EOT goals has been largely accepted by virtue of its low incidence, however rHAT is thought to be significantly underreported, due both to its acute presentation—limiting opportunities for early diagnosis and utility of active screening activities—and absence of the priority status afforded to gHAT control and surveillance, with modeling work estimating up to 12 deaths occurred for every one reported death in an rHAT focus in Uganda [12]. While a stage-independent oral therapeutic was recently approved for gHAT—fexinidazole [13]—for rHAT treatment options remain stage-specific. In rHAT cases that are detected, detection is rarely in the hemolymphatic stage, leaving melarsoprol, a chemotherapeutic agent with an associated fatality of 5% [14], as the only treatment option. Finally, animal African trypanosomiasis (AAT) is an important limiting factor for livestock productivity and poverty alleviation in endemic areas [15], and non-medical control efforts for AAT and HAT coincide. The underreporting of and limited treatment options for rHAT, and the economic importance of AAT and opportunity to coordinate control of both diseases in a One Health framework, suggest that rHAT should not be overlooked.

With regards to gHAT, the Informal Expert Group on *Gambiense* HAT Reservoirs concluded in a recent review that latent human infections and animal reservoirs threaten EOT goals for gHAT, yet little is known of the role of animal reservoirs in the epidemiology of this form. The maintenance of certain gHAT foci at hypo-endemic levels, the finding that human-derived *T. b. gambiense* strains cyclically transmitted between animals for more than a year remained human-infective [16], and the failure of some mathematical models to capture low prevalence dynamics without inclusion of an animal reservoir or invasion of infected tsetse, all suggest animal reservoirs for gHAT exist [11, 17–19]. Due to experimental evidence that *T. b. gambiense* can retain infectivity after repeated passage through pigs and that pigs may remain infected for prolonged durations (up to 18 months), the current consensus is that pigs are the animal reservoir most likely to threaten gHAT elimination [11, 16]. Furthermore, several important gHAT vectors have demonstrated a preference for pigs, who roam in humid and shady areas in the periphery of villages or along small rivers, resulting in high exposure to these riverine tsetse [20].

In this study, we use a time series of high-resolution cattle and pig density maps, HAT surveillance data collated by the WHO Atlas of HAT, and the parametric g-formula [21] to estimate the effect of livestock density on HAT risk. Defining livestock density as the ratio of animals to humans, we have conducted this analysis separately for each country, animal species, and gHAT and rHAT, in Malawi, Uganda, Democratic Republic of the Congo (DRC), and South Sudan, four high-burden countries that do not yet meet eligibility criteria for EPHP validation. While there is ample evidence documenting the reservoir role of cattle in rHAT, the strength of this effect and the corresponding effect for pigs is likely focus-dependent. Country-specific estimates of the livestock effect, in concert with high-resolution data on livestock distribution, will provide both key parameters for rHAT modeling efforts and key inputs for national sleeping sickness control programs and other stakeholders wishing to identify subnational foci where transmission may be going undetected, or where insecticide or trypanocide treatment of domestic animals may achieve EPHP (or even EOT). In gHAT foci, estimation of this effect will narrow critical knowledge gaps surrounding the role of domestic animals in gHAT transmission.

## Materials and methods

Our study is a retrospective ecological cohort study, with cluster-year (0.017° pixel) as the unit of analysis in Malawi, Uganda, and DRC, and county (administrative level 2) as the unit of analysis in South Sudan.

### Study population

Our study population was defined as all subnational locations at risk of reporting a HAT case; i.e., all locations which could generate a case report in the WHO Atlas of HAT, the source of our outcome data. In Uganda, DRC, and Malawi, we defined this study population as all clusters within five hours’ travel time of a fixed health facility capable of HAT diagnosis [22]. In Uganda, where gHAT and rHAT coexist, study areas were defined separately for each form; only gHAT is endemic in DRC, and only rHAT is endemic in Malawi. In South Sudan, where gHAT is endemic, we restricted the study area to counties which reported one or more HAT cases or conducted active surveillance during the study period.

The study period was defined separately for each country, on the basis of WHO Atlas of HAT data access provided to the authors and available exposure data: 2006–2018 for Uganda, 2003–2014 for Malawi, and 2010–2013 for DRC. In South Sudan the study period was restricted to 2008 alone due to limited exposure data availability, as detailed below.

### DAG formulation

**Fig 1:**
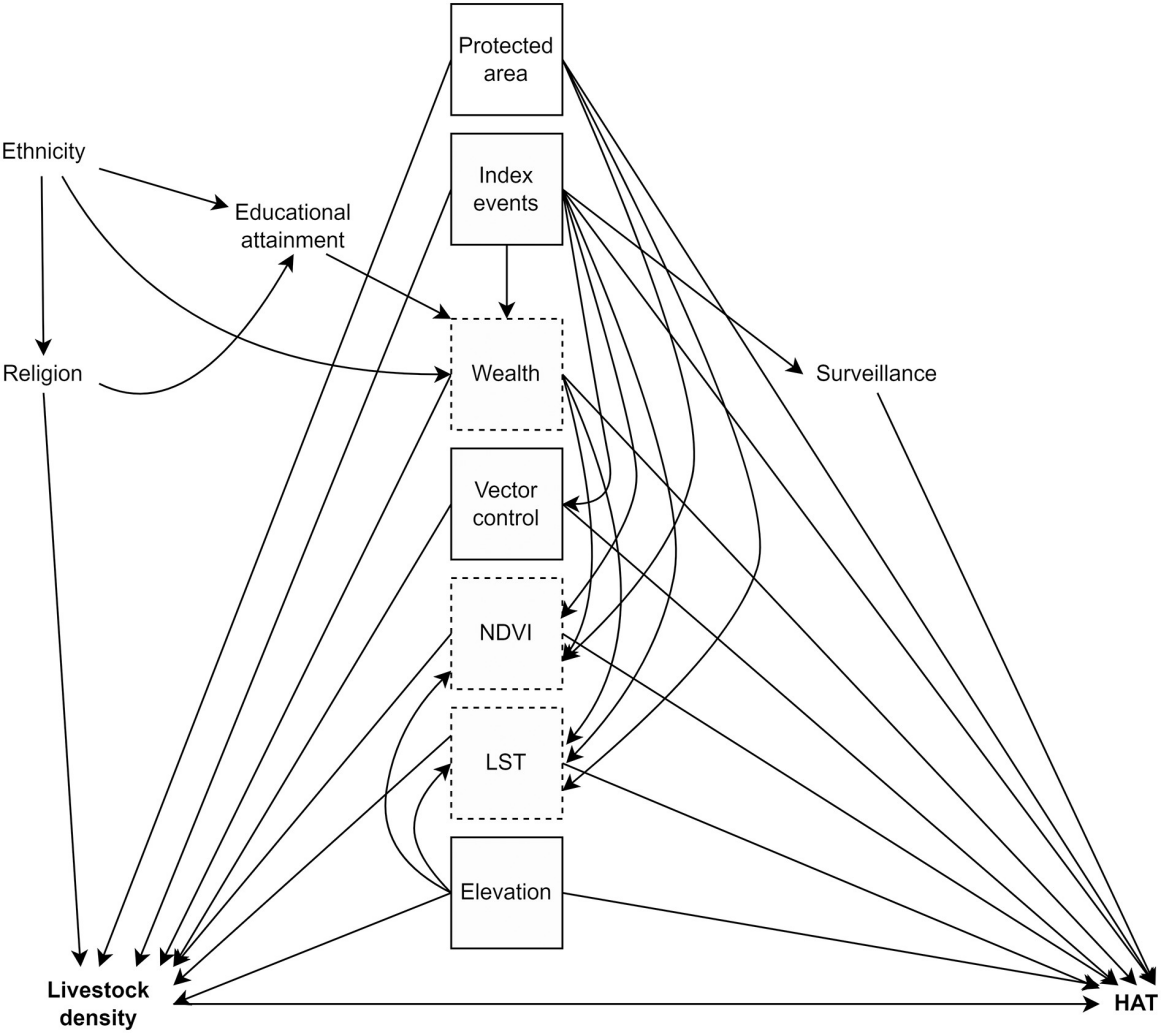
Time-stratified directed acyclic graph (DAG). Boxed variables are those in the minimum sufficient adjustment set; those with a dashed line exhibit exposure-confounder feedback. Bolded variables are the exposure and outcome of interest. Protected areas refers to rHAT models only.

We identified confounders by *a priori* subject knowledge, which we encoded in a directed acyclic graph (DAG). We used an initial time-stratified DAG (Fig 1) and DAGitty.net [23] to identify the minimally sufficient adjustment set.

“Index events” refers to natural and technological disasters and armed conflict. Location within a protected area was considered for rHAT alone, as a proxy for presence of wildlife reservoirs. Note that all variables are cluster-level, representing proportions for variables which are composites of individual-level categorical variables (ethnicity, educational attainment, and religion), and means for composites of continuous variables (wealth). The minimally sufficient adjustment set is {elevation, index events, LST, NDVI, wealth, vector control}. NDVI, or the normalized difference vegetation index, is a remote-sensed indicator of vegetation coverage; LST, or land surface temperature, is a remote-sensed indicator of temperature.

**Fig 2:**
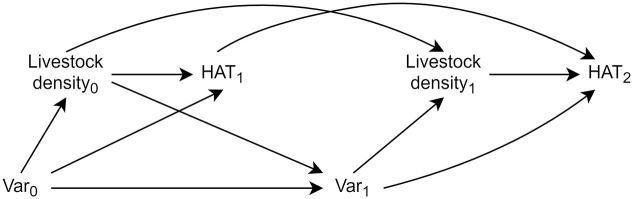
Time-varying directed acyclic graph (DAG) demonstrating exposure-confounder feedback. HAT_1_ and HAT_2_ refer to HAT risk at two time points. Var_0_ is a vector containing wealth, NDVI, and LST at the first of two hypothetical time points, and Var_1_ is a vector containing the same variables at the second time point. Var_1_ is a confounder of the livestock density_1_—HAT_2_ pathway, but is a mediator of the livestock density_0_—HAT_1_ and livestock density_0_—HAT_2_ pathways, thus adjustment of Var={Var_0_, Var_1_} will bias the joint (over time) effect of livestock density on HAT.

The variables marked with a dashed box in Fig 1 exhibit exposure-confounder feedback: they are time-varying variables which are causes of the outcome and are both downstream of earlier exposure (mediators) and upstream of later exposure (confounders) (Fig 2). As unbiased estimation of the total exposure-outcome effect requires adjustment of confounders but precludes adjustment of mediators, traditional regression adjustment cannot be utilized. Robin’s generalized methods, referred to as “g-methods,” allow the identification and estimation of causal effects in the face of time-varying confounding with exposure-confounder feedback. As we do not have longitudinal exposure data in South Sudan, time-varying confounding is not a concern for analysis for this country.

**Fig 3:**
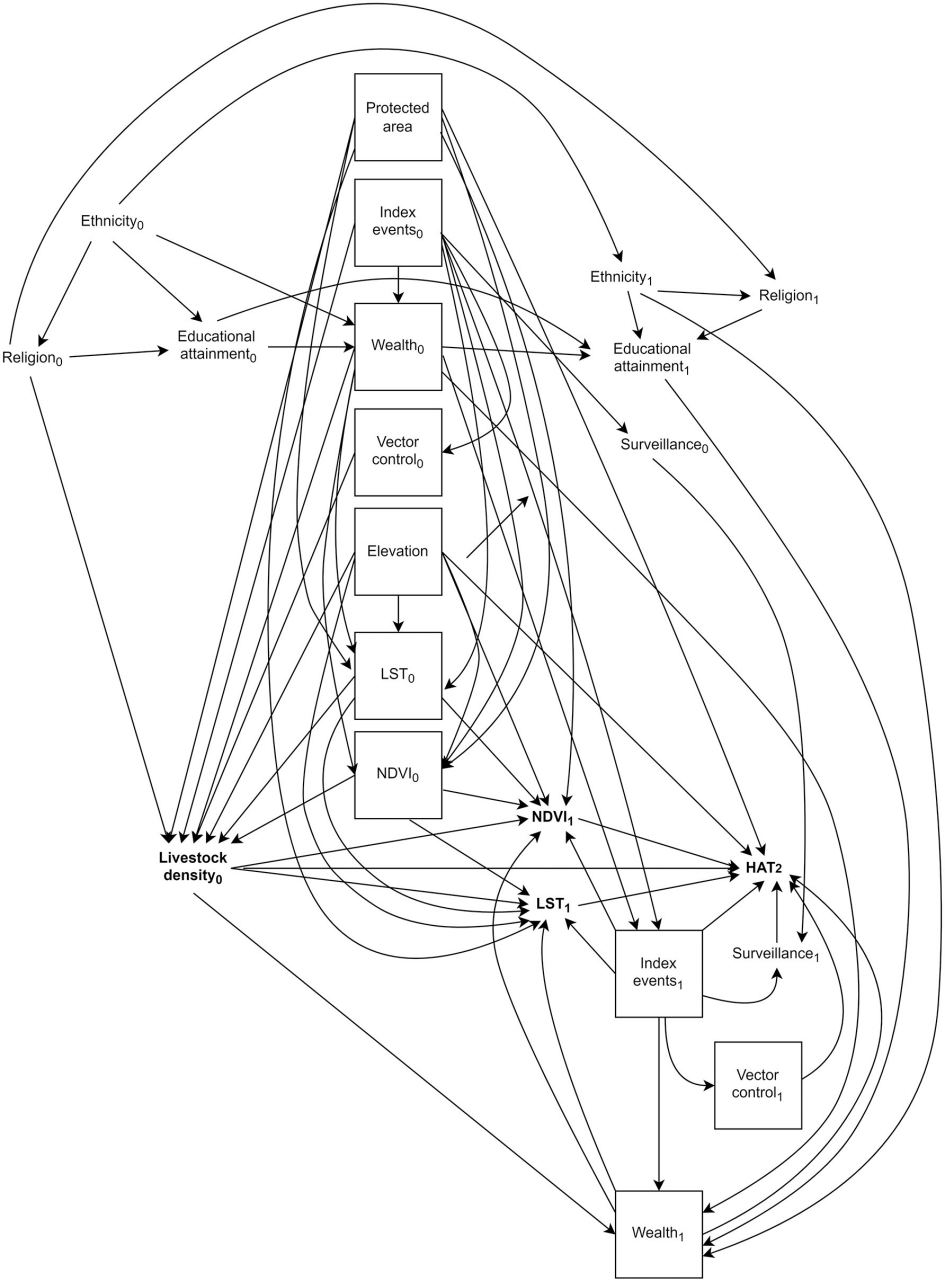
Final time-varying directed acyclic graph (DAG). Protected areas pertains to rHAT models only. Exposure and outcome of interest are bolded, and confounders in the minimally-sufficient set are denoted by solid boxes. HAT_1_ and HAT_2_ refer to HAT risk at two time points.

Our final longitudinal DAG, restricted to the minimally sufficient adjustment set and to three time points, is presented in Fig 3. We assume index events are upstream of environmental variables (LST and NDVI) and wealth measured at the same time, but impose lags of one year for the effects of index events, environmental variables, and wealth on both livestock density and HAT risk.

For all time-varying variables, we assume all effects are completely mediated by the same variable’s value at the subsequent time point; for instance, the effect of index events at time 0 on HAT risk at time 2 is fully mediated by index events at time 1, with no direct effect. The exception to this is vector control, in which case we assume there is neither a direct effect on HAT at greater than one lag (i.e., no directed edge from vector control_0_ to HAT_2_), nor an indirect effect mediated by vector control at the subsequent time (i.e., no directed edge from vector control_0_ to vector control_1_).

### Measures

#### Exposure

Exposure is defined as livestock density, parameterized using a time-series of maps we have created and detailed in a separate publication [24]. We use the term “livestock” to refer collectively to cattle and pigs, however all analyses are conducted separately for each species. Livestock density is defined as the ratio of animals to humans.

For Malawi, Uganda, and DRC we generated continuous maps by year for the study period. We validated these maps using leave one out cross validation, and by comparing our final 2010 maps against the Gridded Livestock of the World 3 maps [25, 26], using WorldPop data to estimate human population and therefore density [27]. For South Sudan, given limited availability of data that provided both livestock and location data, it was only possible to produce areal (county-level) maps, and only for 2008.

#### Denominator data

Denominator data came from the University of Southhampton’s WorldPop project, which produces high resolution (1km at the equator) data on human population distributions using a flexible random forest estimation technique [28].

#### Outcome data

Outcome data included all new cases of HAT diagnosed in a given year and given cluster (Malawi, Uganda, DRC) or county (South Sudan) in the WHO Atlas of HAT [29]. Clusters which were not represented in the Atlas and were at least 1km from any clusters with a reported case were assigned 0 cases.

#### Confounders

We estimated wealth using an exploratory factor analysis approach modeled after the DHS Wealth Index [30] but excluding livestock-related measures. We then used spatial modeling to generate continuous annual maps (detailed in S1 Appendix).

We used the EM-DAT database to identify natural disasters during the study period, and UCDP/PRIO Armed Conflict Database to identify armed conflicts [31]. Disaster was parameterized as a binary variable for our models, taking value = 1 if an event occurred in a given cluster-year or county. Data on NDVI came from the Land Long Term Data Record 5 (LTDR5) [32]. Data on LST came from MODIS/Terra MOD21 for 2000–2002 [33], and from MODIS/Aqua MYD21 for 2003–2014 [34]. Elevation data came from GMTED2010 [35]; for our analyses we used the 7.5-arc-second data, which has a root mean squared error of 26–30 meters, and median elevation.

**Fig 4:**
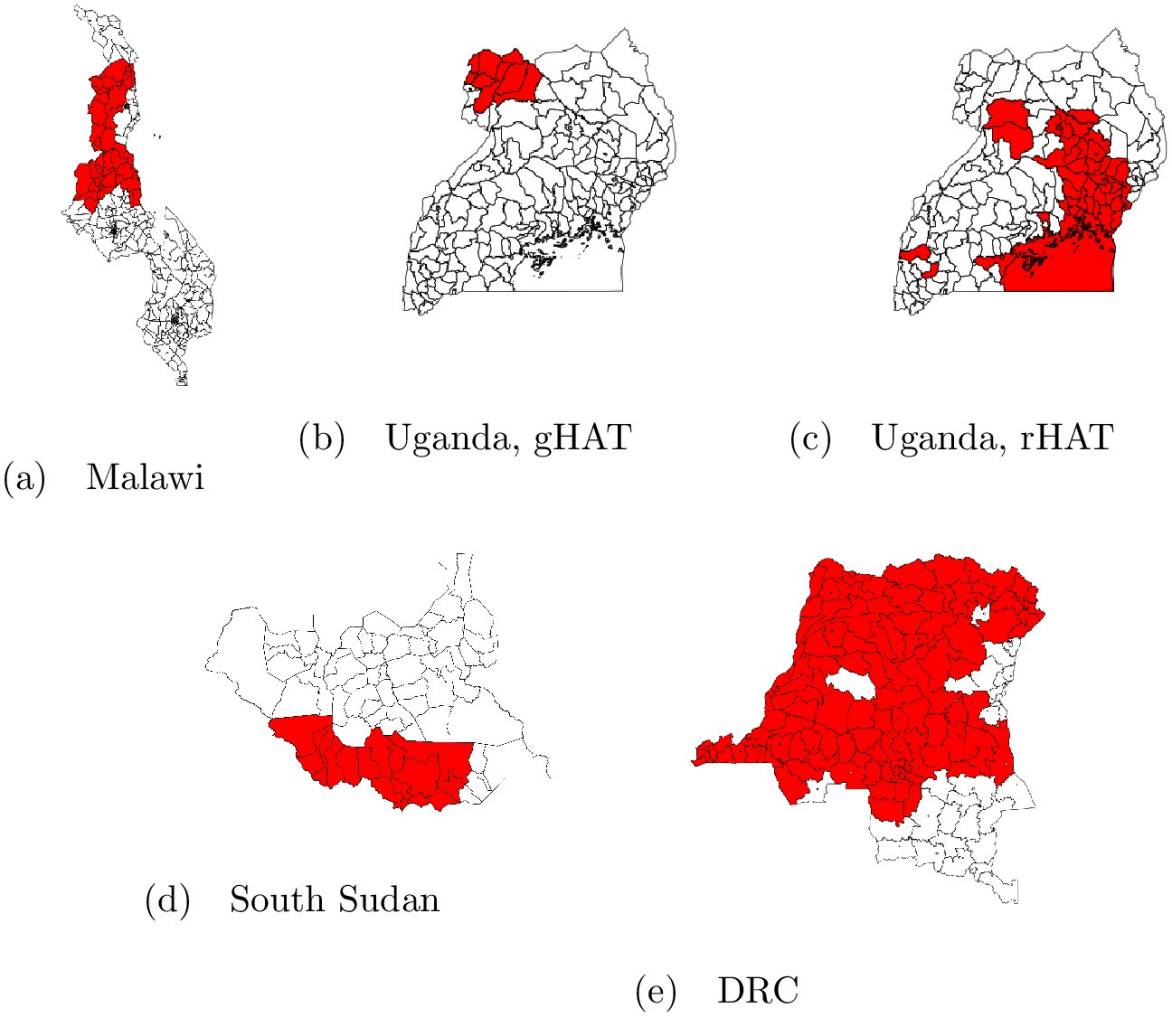
National maps with administrative areas represented in the study data highlighted in red. (a) Malawi: traditional authority (administrative level 3); (b-d) Uganda and South Sudan: county (administrative level 2); (e) DRC: territory (administrative level 2). All base maps were obtained from GADM [47].

We did not attempt to adjust for vector control as it is not practical to parameterize farmer-led efforts, and top-down vector control efforts tend to be deployed across an entire epidemic focus, resulting in no spatial variability within each epidemic modeled and thereby precluding adjustment (e.g., across the entire Uganda gHAT focus modeled in our study [36]; Fig 4). We do, however, present E-values for our results to indicate the level of bias due to unmeasured confounding, for instance by vector control. E-values are defined as the minimum strength of association of uncontrolled confounding (i.e., an unmeasured confounder, or a confounder measured with significant error) that would be required to fully account for the effects detected. These are presented on the relative risk scale [37, 38].

### Parametric g-formula

In Uganda, DRC, and Malawi, we used the parametric g-formula to estimate the total effect of livestock density on HAT risk in the presence of exposure-confounder feedback. Under the potential outcomes framework for causal inference, causal estimands are defined in terms of counterfactual outcomes *Y*^*a*^—specifically, contrasts of these counterfactual outcomes under different exposure values *Y*^*a*^ − *Y*^a^*—where *Y* denotes outcome and *A* = *a* denotes exposure fixed at level *a*. The g-formula applies standardization to estimate counterfactual outcomes, and the parametric g-formula uses parametric models to extend the g-formula to settings with numerous or high-dimensional confounders [39].

While individual-level counterfactual outcomes cannot in general be estimated from observed data, population-average counterfactual outcomes *E*[*Y*^*a*^] can be estimated if three conditions are met: (conditional) exchangeability, positivity, and stable unit treatment value assumption (SUTVA). The identifiability criteria and implementation of the parametric g-formula are detailed in S2 Appendix. Broadly, models are fit for each time-varying confounder and for outcome at each discrete time point, and predictions generated from each model under exposure value *A* = *a*. We defined *A* = a* as mean livestock density, and *A* = *a* as mean × 1.5 and specify our causal estimand on the ratio scale (S2 Appendix), generating a rate ratio analog corresponding to a 50% increase in exposure.

As the no interference assumption is violated in our study, we instead turn to partial interference, in which each unit of observation has a specified interference set *χ*_*i*_ = {*i*_1_, *i*_2_, …} [40, 41]. Partial interference is satisfied if each unit’s potential outcome is independent of the rest conditional on this interference set. Previous authors have defined the interference set in terms of its member’s counterfactual outcomes, however applications to correlation in space have been limited to linear space [41]. In our application, interference arises through movement of tsetse flies, livestock, and people. As grazing radius can vary from less than one to 10 or more kilometers per day, and tsetse flies have a range with a radius of approximately 300 meters per day, we define the interference set as all other clusters within a 5km radius [42, 43].

Validation of the parametric g-formula is conducted through implementation of the “natural course” model, whereby exposure (here, livestock density) is not fixed, but rather modeled as for time-varying confounders. The simulated outcome distribution is then compared against the observed to determine how successfully the observed distribution was recovered.

### Regression

As we did not have longitudinal data in South Sudan, exposure-confounder feedback was not a concern, allowing us to use spatial regression models rather than the parametric g-formula. Specifically, we fit Bayesian hierarchical models with ICAR and iid random effects in space (county), implemented using the R-INLA package [44].

We first fit naive Poisson models which did not account for measurement error in livestock density or wealth—that is, did not acknowledge that these variables were estimated:

$$Y_{c}|\lambda ∼\text{Poisson}\left(\lambda \right)$$

$$E\left[Y_{c}\right]=\lambda P_{c}=exp(\beta_{0}+\beta_{1}\mu_{c}+\alpha z\left(b_{c}\right)+S_{c}+\epsilon_{c}+log\left(P_{c}\right))$$

$$\epsilon_{c}|\sigma_{\epsilon }^{2}{∼}_{iid}N\left(0,\sigma_{\epsilon }^{2}\right)$$

$$S_{c}|S_{k},k\in ne\left(c\right)∼N({\bar{S}}_{k},\frac{\sigma_{s}^{2}}{m_{c}})$$

where

- *Y*_*c*_ is the number of cases in county *c*
- λ is a rate
- *μ*_*c*_ is estimated livestock (cattle or pig) density in county *c*
- ***α*** is a vector of coefficients
- **z**(**b**_*c*_) is a vector of confounders
- *S*_*c*_ are the structured (ICAR) spatial (county) random effects
- *ϵ*_*c*_ are the unstructured (iid) spatial (county) random effects
- *P*_*c*_ is the offset, given as population in county *c*
- *ϵ*_*c*_ are county-level iid (unstructured) random effects with variance $\sigma_{\epsilon }^{2}$
- *S*_*c*_ are county-level structured random effects which follow the ICAR model with marginal variance $\sigma_{s}^{2}$
- *ne*(*c*) denotes neighbors (shared boundary) of county *c*
- *m*_*c*_ is the number of neighbors of county *c*

and ICAR is the intrinsic conditional autoregressive model, which smooths each county’s random effect to that of its neighbors, with more smoothing performed for counties with fewer neighbors. We parameterized exposure (livestock density) such that exponentiated effect estimates could be interpreted as the rate ratio for a 50% increase in density, allowing direct comparison with g-formula results in the other countries. We used penalized complexity (PC) priors for the smoothing model, with *Pr*(*σ*_*ϵ*_) > 1 = *Pr*(*σ*_*s*_) > 1 = 0.01. This yields a posterior 99% credible interval for each random effect’s residual rate ratio of (0.36, 2.71) [45, 46].

Next, we re-fit these models, this time specifying livestock density and wealth as random effects following a classical measurement error (MEC) model (implementing these models within the g-formula was not practical for the other study countries). Under this model, the true value *x* cannot be observed directly, but is instead measured with error. We detail implementation of this model in S3 Appendix.

As a sensitivity analysis, we also repeated these analyses restricted to the active surveillance data, as we expect bias in HAT reporting to follow different mechanisms in the active surveillance versus full dataset.

## Results

### Descriptive statistics

After removing predicted clusters within 1km of locations in the WHO Atlas of HAT data and more than 5 hours’ travel time from fixed health facilities capable of HAT diagnosis, we were left with 3,982 clusters/year in Malawi, 5,746 clusters/year for gHAT models in Uganda, 13,570 clusters/year for rHAT models in Uganda, and 247,205 clusters/year in DRC. In South Sudan, the study area was comprised of 17 counties, with active surveillance being performed in 14 of them in 2008. General study areas are presented in Fig 4. Note this figure represents the administrative areas represented in the study data in each country, however in Malawi, Uganda, and DRC, the study area is defined at the cluster-level, not the areal-level (i.e., not all clusters within a given administrative area highlighted in Fig 4 appear in the study data).

No armed conflicts in Malawi appeared in the UCDP/PRIO database during the study period, and no natural disasters in South Sudan appeared in the EM-DAT database in 2008. Descriptive statistics are presented in Tables 1 and 2, with additional descriptive statistics figures presented in S4 Appendix, including temporal trends (aggregated over clusters) and maps for key variables.

**Table 1:** Descriptive statistics, Malawi, Uganda, and DRC.

Variable	Mean (sd)			
	Malawi	Uganda, gHAT	Uganda, rHAT	DRC
Cattle density	0.09 (0.31)	0.31 (0.92)	0.16 (0.42)	0.06 (0.06)
Pig density	0.09 (0.19)	0.24 (0.72)	0.06 (0.10)	0.06 (0.03)
Elevation (meters)	1,125 (280)	890 (166)	1,099 (79)	524 (194)
HAT cases	0.01 (0.14)	0.04 (0.35)	0.01 (0.15)	0.02 (0.35)
LST (K)	307 (4.7)	281 (96)	277 (95)	306 (3.67)
NDVI	0.15 (0.14)	0.28 (0.13)	0.26 (0.21)	0.22 (0.18)
Wealth	1.30 (1.04)	0.84 (0.03)	0.85 (0.03)	0.25 (0.22)
Conflict	0 (0%)*	743 (0.68%)*	1,824 (0.71%)*	177,468 (9%)*
Disaster				
Flood	19,548 (33%)*	413 (38%)*	2,290 (0.9%)*	46,921 (2.37%)*
Storm	1,213 (2%)*	0 (0%)*	0 (0%)*	0 (0%)*
Epidemic	0 (0%)*	2,065 (1.9%)*	6,307 (2.5%)*	28,343 (1.43%)*
Landslide	0 (0%)*	0 (0%)*	127 (0.05%)*	0 (0%)*
Drought	0 (0%)*	0 (0%)*	8 (<0.01%)*	0 (0%)*
Earthquake	0 (0%)*	0 (0%)*	0 (0%)*	410 (0.02%)*
Wildfire	0 (0%)*	0 (0%)*	0 (0%)*	16,575 (0.84%)*

Descriptive statistics over study clusters and period (2000–2014 for Malawi, 2000–2018 for Uganda and DRC). sd: standard deviation.

*n(%).

Conflict is defined as the number of cluster-years which experienced an armed conflict

**Table 2:** Descriptive statistics, South Sudan.

Variable	Mean (sd)
Cattle density	0.77 (1.15*)
Pig density	0.02 (1.25*)
Elevation (meters)	693 (158)
HAT cases	34 (52)
Number screened	2,953 (4,200)
LST (K)	312.54 (3.22)
NDVI	0.28 (0.07)
Wealth	0.19 (0.004*)
Conflict	8 (47%)**

Descriptive statistics over study counties. 2008 data: cattle density, pig density, HAT cases, number screened, WorldPop population, LandScan population, wealth. 2007 data: LST, NDVI. 2006 data: conflicts. sd: standard deviation.

*Mean of design-based standard error.

**n, (%).

Conflict is defined as the number of counties which experienced an armed conflict in 2006

### Parametric g-formula

In Uganda, for the rHAT models the mean of the squared difference between observed HAT cases and that predicted by the “natural course” model across cluster-years was less than 1 x 10^−4^ for both cattle and pigs. For the gHAT models, the pig models performed very badly in 2010 and 2012; removing these years from all subsequent analyses for pigs, this value was < 0.0005 for both cattle and pigs. In Malawi and DRC, this value was < 1 × 10^−4^ for both species across all years.

In the Malawi models, several confounders needed to be dropped due to the very low number of observed cases (as low as 18 cases reported in 2012) and resulting concerns surrounding model over-fitting. Namely, location within a protected area, elevation, and disasters were removed from the Malawi models. Furthermore, LST was dropped from all analyses due to very high levels of missingness.

Results from the parametric g-formula are presented in Table 3. We found a 50% increase in cattle density was associated with a 61% higher risk of rHAT in Uganda (95% CI 0.90, 2.99) and a 12% lower risk of rHAT in Malawi (95% CI 0.71, 1.10). For gHAT, a 50% increase in cattle density was associated with a 12% lower risk of gHAT in Uganda (95% CI 0.50, 1.77) and a 17% higher risk of gHAT in DRC (95% CI 1.04, 1.32). For pigs, a 50% increase in density was associated with a 107% higher rHAT risk in Uganda (95% CI 1.15, 2.75) and a 58% lower rHAT risk in Malawi (95% CI 0.23, 0.67). For gHAT, a 50% increase in pig density was associated with a 102% higher risk of gHAT in Uganda (95% CI 0.87, 3.94) and a 23% higher risk of gHAT in DRC (95% CI 1.10, 1.37). These effects did not reach significance for the cattle-rHAT effect in Uganda or Malawi, or the cattle-gHAT and pig-gHAT effects in Uganda.

**Table 3:** Parametric g-formula results.

	RR (95% CI)		
	Uganda	Malawi	DRC
**Cattle**			
rHAT	1.61 (0.90, 2.99)	0.88 (0.71, 1.10)	-
gHAT	0.88 (0.50, 1.77)	-	1.17 (1.04, 1.32)
**Pigs**			
rHAT	2.07 (1.15, 2.75)	0.42 (0.23, 0.67)	-
gHAT	2.02 (0.87, 3.94)	-	1.23 (1.10, 1.37)

Parametric g-formula implemented such that effect estimates correspond to a 50% increase in livestock density. Malawi results are for rHAT only, and DRC results are for gHAT only. RR: rate ratio analog; 95% CI: credible interval over 100 iterations of the parametric g-formula

### Regression

Total effect estimates are presented in Table 4. While both results are presented, we will only discuss results from the measurement error model as the naive model estimates are biased. After adjustment for confounders {wealth, NDVI, LST, elevation, armed conflict}, the rate ratio (RR) was 0.63 (95% CI 0.54, 0.77) for cattle, and 0.66 (0.50, 0.98) for pigs. Cattle effect estimates were slightly attenuated and no longer significant when restricted to active surveillance data (RR 0.79, 95% CI 0.35, 1.20), while pig estimates were not appreciably changed (RR 0.59, 95% CI 0.36, 0.91).

**Table 4:** Total effect results, South Sudan.

Denominator	Model	RR (95% CI)
**Cattle**		
WorldPop	Naive	0.59 (0.37, 0.83)
WorldPop	MEC	0.63 (0.54, 0.77)
Number sampled	Active surveillance	0.79 (0.35, 1.20)
**Pigs**		
WorldPop	Naive	0.63 (0.31, 1.18)
WorldPop	MEC	0.66 (0.50, 0.98)
Number sampled	Active surveillance	0.59 (0.36, 0.91)

Posterior mean rate ratios and 95% credible intervals for livestock density in South Sudan, after adjustment for wealth, NDVI (lagged 1 year), LST (lagged 1 year), elevation, and armed conflict. Density is parameterized such that rate ratios correspond to a 50% increase in density. RR: rate ratio; CI: credible interval; Naive: models which do not account for measurement error in wealth or livestock density; MEC: measurement error models

### E-values

E-values are presented in Table 5. In all cases, for unmeasured confounding to be the sole explanation for our findings (c.f. a true livestock-HAT effect), this unmeasured confounding would need to be very strong. This is particularly true for the cattle-rHAT association in Uganda, the pig-rHAT association in Uganda and Malawi, the pig-gHAT association in Uganda and South Sudan, and the cattle-gHAT association in South Sudan.

**Table 5:** E-value results.

	Uganda	Malawi	DRC	South Sudan
**Cattle**				
rHAT	2.6	1.53	-	-
gHAT	1.53	-	1.62	2.55
**Pigs**				
rHAT	3.56	4.19	-	-
gHAT	3.46	-	1.76	2.4

The strength, on the relative risk or rate ratio scale, of the relationship between an unmeasured confounder and exposure (livestock density) and between an unmeasured confounder and outcome (HAT risk) that would be required to fully explain the estimated effect. South Sudan results are presented for measurement error models.

### Simulations

While the finding of a protective effect in Malawi for both cattle and pigs (albeit non-significant) has several possible explanations other than bias—detailed below in our Discussion section—bias due to failure of passive surveillance systems resulting in underreporting is a significant concern. Similarly, the finding of a possible reservoir effect of cattle for gHAT in DRC is surprising and inconsistent with the gHAT findings in Uganda and South Sudan, and may be explained by underreporting due to failure of passive surveillance systems, or to undercoverage of active surveillance systems in high-risk foci, for instance if mobile teams cannot access a location for security or logistical reasons.

We therefore conducted a simulation study to explore the degree of underreporting that would be needed to “explain away” these findings: effectively, the underreporting level at which the entirety of the effect we detected could be attributed to underreporting. In Malawi, all scenarios followed a model in which a 50% increase in cattle density causes a 20% increase in rHAT risk, and the same increase in pig density causes a 15% increase in risk (corresponding to a relative risk of 1.2 for cattle and 1.15 for pigs, with exposure parameterized equivalently to our main results). Test scenarios differed by the level of underreporting: 0.5, 3, and 10 unreported cases per 1 reported case. For DRC, all scenarios followed a model in which a 50% increase in cattle density causes a 20% decrease in gHAT risk, and the same increase in pig density causes a 15% decrease in gHAT risk (corresponding to a relative risk of 0.8 for cattle, and 0.85 for pigs). Underreporting levels were simulated at 0.1, 0.3, and 0.5 unreported cases per 1 reported case. We simulated a relatively higher underreporting level in Malawi than DRC (i.e., a max of 10 unreported cases per reported cases in Malawi, versus a max of 0.5 unreported cases per reported case in DRC) due to the absence of active surveillance activities in Malawi and evidence that underreporting is much higher for rHAT than gHAT foci.

Results are presented in Table 6. We found that under the modeled scenarios, a relatively high level of underreporting would be required to explain our results in these two countries. In Malawi, if indeed cattle play a reservoir role among the unreported cases (contrary to our findings), there would need be 3 or more unreported cases per 1 reported case for our analysis to erroneously detect a protective effect; for pigs, an even higher level of underreporting would be required. In DRC, negative (protective) effects were not recovered under any of the simulated levels of underreporting, indicating our findings cannot be explained by this form of bias at the simulated levels. Note that in Malawi the highest level of underreporting corresponds to 4,122 unreported cases, while in DRC this level corresponds to 18,135 unreported cases.

**Table 6:** Simulation results.

RR (95% CI)		
Underreporting ratio	Cattle	Pigs
**Malawi**		
0.5	0.94 (0.77, 1.09)	0.55 (0.36, 0.87)
3	1.03 (0.91, 1.16)	0.92 (0.62, 1.36)
10	1.19 (1.10, 1.32)	1.49 (1.06, 2.05)
**DRC**		
0.1	1.14 (1.03, 1.26)	1.01 (0.99, 1.06)
0.3	1.17 (1.07, 1.25)	1.03 (1.00, 1.11)
0.5	1.17 (1.08, 1.27)	1.04 (1.00, 1.11)

Results from implementation of the parametric g-formula under a variety of simulated underreporting scenarios, each implemented such that effect estimates correspond to a 50% increase in livestock density. Underreporting ratio calculated as # unreported cases / # reported cases. Malawi results are for rHAT only, and DRC results are for gHAT only. RR: rate ratio analog; 95% CI: credible interval over 100 iterations of the parametric g-formula.

## Discussion

In Uganda we found cattle (RR 1.61, 95% CI 0.90, 2.99) and pigs (RR 2.07, 95% CI 1.15, 2.75) both have a positive effect on rHAT risk, however this effect was significant for pigs only. For gHAT, we found cattle have negative (protective) effect (RR 0.88, 95% CI 0.50, 1.77), while pigs have a positive (harmful) effect (RR 2.02, 95% CI 0.87, 3.94), with neither effect reaching significance. In DRC, we detected a positive effect on gHAT for both cattle (RR 1.17, 95% CI 1.04, 1.32) and pigs (RR 1.23, 95% CI 1.10, 1.37), being marginally stronger for pigs than for cattle; both results were statistically significant.

In Malawi (rHAT) and South Sudan (gHAT), we found both cattle (Malawi: RR 0.88, 95% CI 0.71, 1.10; South Sudan: RR 0.63, 95% CI 0.54, 0.77) and pigs (Malawi: RR 0.42, 95% CI 0.23, 0.67; South Sudan: RR 0.66, 95% CI 0.50, 0.98) have protective (negative) effects, with this result being stronger in pigs than in cattle in Malawi and cattle results not reaching statistical significance in this country. In South Sudan, restricting analyses to the active surveillance data did not appreciably change our results.

These results indicate the zooprophylactic effect—whereby livestock exert a protective effect on HAT risk due to tsetse preference for animal hosts over humans—outweighs the reservoir effect for both cattle and pigs in Malawi and South Sudan and for cattle in gHAT foci in Uganda, however the reservoir effect outweighs the zooprophylactic effect for pigs in gHAT foci in both DRC and Uganda, for cattle in gHAT foci in DRC, and for pigs and cattle in rHAT foci in Uganda. Comparison of results across cattle versus pigs should also consider our choice of a relative parameterization for exposure; that is, a 50% increase in pig density is not equivalent, in absolute terms, to a 50% increase in cattle density, within and across foci. It is important to note that the detected reservoir effects do not necessarily imply that livestock are a maintenance reservoir for gHAT (i.e., sufficient to maintain transmission in the absence of humans), though this is possible.

Note, we have not modeled any interactions (effect modification), for instance between cattle density and pig density. Thus if the cattle density-HAT effect is modified by pig density, our estimated effects can be interpreted as a weighted average of stratified effects. For instance, if cattle and pigs are both reservoirs and thus their co-existence amplifies HAT risk up to a threshold, beyond which they exist in competition, the estimated cattle-HAT effect is essentially a weighted average of a series of cattle-HAT effects stratified by pig density. Similarly, subnational heterogeneity in effects, for instance due to vector species, vector behavior, vector control, or other determinants of host-vector contact, is not captured as we have fit one model per country (with the exception of Uganda) without interaction by more granular location (e.g., district). Thus the estimated effects are a weighted average of location-specific effects. Note this does not preclude coupling country-level effects with location-specific estimates of livestock density for subnational deployment of control efforts.

Beyond interactions, threshold effects and other non-linearities in the livestock density-HAT effect will drive model misspecification, though note the g-formula does allow the livestock density-HAT effect to change over time (i.e., flexibility in temporal effects). If these non-linearities exist, our estimated effects will reflect first-order (linear) trends in the livestock density-HAT effect, rather than absolute truths.

Our findings initially appear discordant with mechanistic knowledge about HAT transmission, i.e., cattle and pigs are reservoirs for rHAT, and as the gHAT focus in Uganda is contiguous with that in South Sudan, transmission dynamics should be nearly equivalent. However, it is important to remember these results reflect complex epidemiological factors that extend beyond transmission mechanisms to local population dynamics and control measures. For instance our Malawi results do not conflict with a scenario in which cattle and pigs are theoretical reservoirs of rHAT, however the wildlife reservoir so markedly dominates these domestic animal reservoirs that the zooprophylactic effect prevails. This could arise if the predominant vector species (*Glossina morsitans morsitans*) exhibits a strong preference for wildlife, or if domestic animal reservoirs are adequately controlled through use of insecticides or trypanocides, or environmental measures such as brush clearing. Similarly, differences in host abundance and control measures between Uganda and South Sudan could explain the discrepancies between the results observed in these two countries. This is particularly relevant for the case of pigs; as South Sudan is a predominantly Muslim country, very few pigs are kept, and the sociocultural characteristics of higher pig density clusters are likely highly divergent from those in low pig density clusters, potentially driving similar divergence in HAT epidemiology. Presence of a zooprophylactic effect in South Sudan and for cattle in gHAT foci in Uganda is consistent with evidence from southeastern Uganda that *Glossina fuscipes fuscipes*, the predominant gHAT vector in South Sudan and Uganda, exhibits strong zoophilic behavior [48]. Note that while there are several possible mechanisms which could give rise to this biting preference (i.e., relative abundance of host species, preferences inherent to fly species, etc.), pointing to different opportunities for intervention, all are expected to result in a net negative effect of livestock density on HAT risk. The finding of a positive cattle-gHAT effect in DRC but not Uganda or South Sudan is surprising, and of uncertain significance.

However, our results could, of course, be subject to bias. With regards to confounding, in Uganda, DRC, and South Sudan we feel we have achieved reasonable control for all confounders except vector control efforts. We would expect vector control to have a positive effect on livestock density (as AAT control would increase livestock density) and a negative effect on HAT risk (through decreased cases), thus unmeasured confounding by vector control would bias effect estimates towards zero, making it harder to detect positive (>1) effects, and easier to detect negative (<1) effects. That is, uncontrolled confounding by vector control would obscure a true reservoir effect, not induce an artificial effect. However, it is possible that failure to adjust for vector control explains the negative effects we are attributing to zooprophylaxis. This zooprophylactic effect was most pronounced in foci with limited top-down vector control efforts (Malawi and South Sudan), but farmer-led efforts cannot be ruled-out as an explanation for this finding. Thus this effect may represent functional zooprophylaxis rather than mechanistic zooprophylaxis, i.e., due to use of trypanocides and/or pesticides on livestock rather than solely tsetse preference for biting livestock. Our calculated E-values also indicate that unmeasured confounding by vector control may explain the rHAT effect for cattle in Malawi and the gHAT effect for cattle in Uganda (effects we have attributed to zooprophylaxis), but is unlikely to be responsible for the other effect estimates. Furthermore, we approximated wealth, a latent construct, using factor analysis (detailed in S1 Appendix), which may not capture the dimension of wealth which is most relevant to our study, driving residual confounding by this variable. Finally, in Malawi we did not adjust for several known confounders due to overfitting concerns. Again, our E-values indicate this unmeasured confounding may explain the cattle results in Malawi, but are unlikely to explain the pig results.

A second major source of bias is selection bias. In Malawi, Uganda, and DRC, study area was defined by proximity to a fixed health facility capable of HAT diagnosis, however we did not have longitudinal data for this variable. In South Sudan, we defined study counties by appearance in the WHO Atlas of HAT dataset, and thus underreporting of the outcome (discussed below) may drive selection bias. In both cases, selection bias arises if excluded clusters or counties differ systematically in their distribution of livestock density or confounders from the included counties or clusters. While we attempted to evaluate the underreporting as the driver of our more surprising findings in Malawi and DRC via simulation studies, the simulated scenarios were somewhat simple, governing only the mechanism by which unreported cases were generated.

As with all research, measurement error threatens the validity of these findings. We attempted to account for measurement error arising from estimation of wealth and livestock density in South Sudan, however these measurement error models do not account for errors in the raw data used for livestock or wealth mapping, for instance a systematic under-reporting of livestock ownership due to concerns about increased taxation. Furthermore, we did not implement measurement error models in the remaining study countries, however we did restrict analyses to years bounded by those with available input data used to generate livestock density estimates [24]. We expect such measurement error to be non-differential with respect to HAT risk, biasing our effect estimates towards the null in expectation. With regards to LST, NDVI, index events, and elevation, measurement error either in the attribute itself, or in its geolocation, will drive residual confounding and bias our effect estimates.

We also expect measurement error in the outcome. As with all surveillance data, reporting of HAT cases is likely to be incomplete, and some HAT foci have no effective surveillance systems [10]. This is particularly true for data collected via passive surveillance, which accounts for all of the Malawi data and Uganda rHAT data. Under-reporting in the passive surveillance data arises from cases who do not seek care, seek care but are not correctly diagnosed, or are diagnosed but not reported [49]. Under-reporting in active surveillance data arises from lack of surveillance in remote or politically unstable foci [50], and incomplete coverage of populations in which active surveillance occurs [51]. If under-reporting is random with regards to space and either HAT risk or livestock density, power will be compromised but effect estimates will be unbiased in expectation. As we expect comparatively less underreporting in the active surveillance data than the passive surveillance data, the fact that our findings in South Sudan were not markedly different when restricted to active surveillance cases alone is reassuring.

Ecological bias may also compromise the validity of our findings. Cross-level (ecological) bias may result from (1) an unmeasured variable which is either (a) an individual-level risk factor whose association with exposure differs across groups, or (b) an individual-level effect modifier whose distribution or effect differs across groups [52]; or (2) aggregation of variables (e.g., from the household to the cluster) [53, 54], termed “pure specification bias” [55]. While wealth was the only confounder measured at the household-level, elevation, NDVI and LST were aggregated from the resolution at which they were defined in the source data to our unit of analysis. Furthermore, analysis of contextual (inherently group-level) variables is not immune to ecological bias [56]. As the size of the ecological association is sensitive to the level of aggregation (being attenuated at higher levels of aggregation), we expect our South Sudan results to be more sensitive to this source of bias than those from Malawi, Uganda, and DRC [57]. Furthermore, findings from ecological studies are sensitive to the grouping definition used [56]; in Malawi, Uganda, and DRC grouping was uniform across space (defined by grid cells), however in South Sudan we utilized administrative boundaries to define groups, which are uneven and do not have meaning to our research question.

Finally, our results are subject to the g-null paradox. When the parametric g-formula is implemented using non-saturated models and the vector of confounders has any discrete components, as is the case in our study, not all models can be correctly specified. While this may result in the null hypothesis being falsely rejected in theory, in practice the g-formula has been able to estimate null effects, indicating the bias induced by the g-null paradox is relatively small compared with random variance [58].

## Conclusion

Our greatest concerns for the validity of these findings are residual confounding by vector control, overfitting of the Malawi models, ecological bias in the South Sudan results, and underreporting in the outcome data. Despite these weaknesses, our study has taken a novel approach to studying the impact of livestock density on HAT risk in a real-world context in which transmission mechanisms exert their effects in the face of spatially-dynamic intervention deployment and vector ecology and behavior, suggesting domestic animals may be important reservoirs of rHAT in Uganda but not Malawi, and pigs—and possibly cattle—may be reservoirs for gHAT. While achieving 2020 targets for EPHP had previously appeared within reach, lapses in control activities due to COVID-19, and long-standing concerns regarding persistent foci and cryptic animal reservoirs, render the need to close HAT knowledge gaps addressed by this research as pressing as ever.

## Supporting information

S1 Appendix Wealth mapping. Methods and results.
(PDF)

S2 Appendix Motivation and implementation of the parametric g-formula. (PDF)

S3 Appendix Implementation of the measurement error model in South Sudan. (PDF)

S4 Appendix Descriptive statistics figures. (PDF)
