# Supplementary material for: The effect of livestock density on Trypanosoma brucei gambiense and T. b. rhodesiense: A causal inference-based approach
Source: PLoS Negl Trop Dis. 2022 Aug 29;16(8):e0010155. doi: 10.1371/journal.pntd.0010155 (PMC9462671; doi:10.1371/journal.pntd.0010155)
Supplement: S1 Appendix — Methods and results. (PDF) [file pntd.0010155.s001.pdf]

S1 Appendix: Wealth mapping

Data collection and processing

Data sources used for livestock and wealth mapping are presented in Table 1.1. There were 10,330 clusters in the final dataset for Malawi, 4,361 in Uganda, and 1,164 in DRC. After reading in data, we harmonized variables so that the wealth index could be calculated across surveys (as detailed in the following section), allowing for smoothing in time.

Table 1.1: Data sources by country

Country	Year	Source
Malawi	2000	Demographic and Health Survey [1]
	2003	World Health Survey [2]
	2004	Second Integrated Household Survey [3]
	2004-2005	Demographic and Health Survey [4]
	2006	Technology Adoption and Risk Initiative Survey [5]
	2010	Demographic and Health Survey [6]
	2010-2011	Third Integrated Household Survey [7]
	2013, 2016	Integrated Household Panel Survey [8, 9]
	2012	Malaria Indicator Survey [10]
	2013-2014	Multiple Indicator Cluster Survey [11]
	2014	Malaria Indicator Survey [12]
	2015-2016	Demographic and Health Survey [13]
	2016-2017	Fourth Integrated Household Survey [14]
	2017	Malaria Indicator Survey [15]
South Sudan	2008	Population and Housing Census [16]
DRC	2007	Demographic and Health Survey [17]
	2010	Multiple Indicator Cluster Survey [11]
	2013-2014	Demographic and Health Survey [18]

Table 1.1: Data sources by country

Country	Year	Source
Uganda	2001	Demographic and Health Survey [19]
	2006	Demographic and Health Survey [20]
	2009	Malaria Indicator Survey [21]
	2009-2010	National Panel Survey [22]
	2010-2011	National Panel Survey [22]
	2011	AIDS Indicator Survey [23]
	2011	Demographic and Health Survey [24]
	2011-2012	National Panel Survey [22]
	2014-2015	Malaria Indicator Survey [25]
	2016	Demographic and Health Survey [26]
	2018	Malaria Indicator Survey [27]

Generating the wealth index

Our process for wealth mapping followed that laid out in the DHS Wealth Index [28] handbook, with the exception of exclusion of livestock-related variables in our wealth index. The final variables included in our wealth models were, defined at the level of the household (M: Malawi, U: Uganda, S: South Sudan, D: DRC):

- Presence of a domestic servant *(M, U, D)*
- Ownership of agricultural land *(M, U, S, D)*
- Amount of land owned (converted to acres) *(M, U, S, D)*
- Ownership of dwelling *(M, U, S, D)*
- Type of fuel used for cooking *(M, U, S, D)*
- Water source *(M, U, S, D)*
- Toilet type *(M, U, S, D)*
- Whether toilet is shared with other households *(M, U, D)*

- Main floor material of dwelling *(M, U, D)*
- Main material of dwelling walls *(M, U, D)*
- Roof material *(M, U, D)*
- Presence of electricity *(M, U, D)*
- One or more household members has a bank account*(M, U, D)*
- Vehicle (none; animal cart or bicycle; motorcycle or scooter; car, mini-bus or truck/lorry) *(M, U, D)*
- Number of rooms per household member *(M, U, S, D)*
- Household owns one or more:
  - Chairs *(M, U, D)*
  - Table *(M, U)*
  - Clock or watch *(M, U, D)*
  - Bucket *(M)*
  - Clothes washing machine *(M)*
  - Dish washing machine *(M, U, D)*
  - Refrigerator *(M, U, S, D)*
  - Mobile phone *(M, U, S, D)*
  - Landline (non-mobile phone) *(M, U, S, D)*
  - Television *(M, U, S, D)*
  - Computer *(M, U, S, D)*
  - Radio *(M, U, S, D)*
  - Sewing machine *(M, D)*
  - Bed *(M, U, D)*
  - Upholstered couch, sofa, chairs, or set *(M, U)*
  - Paraffin or kerosene lamp *(M, U, D)*
  - Boat *(M, U, S, D)*
  - Fishing net *(M)*
  - Mortar and pestle *(M)*
  - Fan *(M, S*
  - Air conditioning *(M)*
  - Machine to play cassette tapes, CDs, DVDs, or Hi-Fi *(M, U)*

- VCR *(Ms)*
- Kerosene or paraffin stove *(M)*
- Electric or gas stove/hot plate *(M, D)*
- Beer-brewing drum *(M)*
- Coffee table for sittingroom *(M)*
- Cupboards, drawers, bureau *(M, U)*
- Desk *(M)*
- Iron for pressing clothes *(M)*
- Satellite dish *(M, S)*
- Solar panel *(M, U)*
- Generator *(M, U, D)*
- Plough *(M)*
- Tractor *(S)*
- Axe or hoe *(D)*

• Proportion of possessions enumerated by the survey in question that the household owns *(M, U, D)*

We coded all variables such that a lower level corresponded to lower wealth, and a higher lever corresponded to greater wealth (e.g., we used the variable number of rooms per household member rather than number of household members per room, as the latter would have a negative association with wealth). We assigned levels to categorical variables as detailed in Tables 1.2-1.7; note apparent repetition within variable levels reflect different categorizations across surveys.

Factor analysis For Malawi, Uganda, and DRC, we performed exploratory factor analysis separately for each of three periods, to reflect the expectation that factor loadings would change over time (e.g., in early periods ownership of a mobile phone would be much more strongly associated with wealth than in later periods): 2000-2006, 2006-2012, and 2012-2020. For South Sudan, data were only available at the county-level and for a single year (2008), thus wealth mapping was conducted at the county-level for 2008.

First, we removed all variables with more than 5% missingness, and all variables with standard deviation equal to 0. We then created a correlation matrix using the `mixedCor`

Table 1.2: Levels for cooking fuel

Level	Fuel types
	Malawi, Uganda., DRC
4	Electricity
3	Gas, kerosene, liquefied petroleum gas, biogas, natural gas, paraffin, oil, solar
2	Charcoal, coal, lignite
1	Wood, firewood (collected or purchased)
0	Dung, straw, shrub, grass, crop residue, saw dust
	South Sudan
4	Electricity
3	Gas
2	Charcoal
1	Firewood
0	Cow dung, Grass

Table 1.3: Levels for water source

Malawi, Uganda, DRC	
Level	Water source
4	Piped into own dwelling, compound, yard or plot
3	Public or communal standpipe/tap, piped into neighbor's dwelling
2	Private (in own dwelling, yard or plot) or public protected well, tube well, borehole, protected spring, rain tank or rainwater, channeled by gravity flow scheme
1	Tanker truck/bowser, cart with small tank, bottled or sachet water, water vendor
0	Unprotected well or spring, private (in own dwelling, yard or plot) or public open well, river, dam, lake, pond, stream, canal, irrigation channel
South Sudan	
3	Water filtering stations with common standpipe, mechanical boreholes with common standpipe, sand filter with common standpipe,
2	Deep borehole with network, deep borehole without network, hand pump, water vendor from deep borehole
1	Shallow well, hafeer/dam with filter, water vendor from shallow well/pond/river/spring
0	Hafeer/dam without filter, still (turda/fula/river) or running (river/pond/tura'a) open water source

Table 1.4: Levels for toilet type

Level	Toilet type
	Malawi, Uganda, DRC
3	Flush toilet (piped, pour flush, or unspecified)
2	VIP latrine, composting toilet, Ecosan toilet, latrine with slab and/or roof
1	Latrine without slab or roof, public pit toilet or latrine, bucket, hanging toilet
0	No facilities/open, bush, field
	South Sudan
5	Private flush toilet
4	Shared flush toilet
3	Private pit latrine
2	Shared pit latrine
1	Bucket
0	No facilities

Table 1.5: Levels for dwelling floor

Level	Floor material
	Malawi, Uganda, DRC
2	Carpet, parquet/polished wood, ceramic or other tile or mosaic, cement/concrete, smooth cement, vinyl/asphalt strips, linoleum, stone, bricks
1	Palm/bamboo, wood planks, broken bricks
0	Earth, sand, dung, smoothed mud

Table 1.6: Levels for dwelling walls

Level	Wall material
	Malawi, Uganda, DRC
3	Cement, concrete, stone with lime/cement or mud, finished/burnt brick with stone or mud, covered adobe
2	Wood, wood planks/shingles, reused wood, plywood, plastic sheet, metal sheet, corrugated iron
1	Mudbrick, unburnt bricks with plaster, cement, or mud, uncovered adobe
0	Thatch, straw, grass, cane/palm/trunks, bamboo or poles with mud, timber, mud, dirt, compacted earth, cardboard, none

Table 1.7: Levels for dwelling roof

Level	Roof material
	Malawi, Uganda, DRC
3	Roofing shingles, ceramic tiles, clay tiles, cement, concrete, calamine/cement fiber, wood, asbsetos
2	Iron sheets, metal, tin
1	Wood planks, cardboard, palm/bamboo grass, rustic mat, plastic/polythene sheeting, tin cans
0	Thatch/palm leaf, grass, sod, mud/earth, none

function in the `psych` package in R [29], and performed exploratory factor analysis using the `fa()` function in the same package, setting our arguments to those specified by the DHS Wealth Index manual: principal components extraction with one factor extracted, imputation of mean for missing data, and estimation of the factor scores using the regression method.

We performed linear regression of the extracted scores on input variables as a sanity check. For all four countries, in general only variables with very large levels of missingness had negative associations with our final wealth score. In all countries, ownership of land was negatively associated with our final wealth score and had high levels of missingness (17% missing in Malawi, 25% in South Sudan, 15% in Uganda, and 31% in DRC). Similar results were found for ownership of dwelling (negative association in Malawi with 87% missing, South Sudan with 0% missing, DRC with 93% missing), area of land owned (negative association in South Sudan with 25% missing, Uganda with 92% missing, and DRC with 93% missing), and household sharing toilet with other households (negative association in Uganda with 12% missing, DRC with 15% missing). In DRC, negative associations were also found for ownership of a canoe, axe, or hoe, with 0% missing for these variables, reflecting absence of a strong association between ownership of these items and wealth. All other variables had positive associations with the final wealth score.

As the goal was to adjust for wealth score in final regression models, we did not perform factor analysis separately for urban and rural clusters in Malawi, Uganda, or DRC, however we use urban/rural status as a predictor in our models (discussed in the Mapping section, below) to reflect both the expected association between this variable and wealth score, and the stratified design of the surveys we used.

Mapping

We detail our approach to livestock mapping in Malawi, Uganda, DRC, and South Sudan using these data in an accompanying publication, available as a preprint in S1 File. This file contains details on the stochastic partial differential equations (SPDE) to Gaussian process modeling adopted in countries with point-level data (Malawi, Uganda, and DRC), and the small area estimation approach adopted in South Sudan.

Malawi, Uganda and DRC After the wealth index was constructed, we collapsed over cluster by taking the mean wealth score in each cluster.

Our predictors included urban/rural status to reflect sampling strategy, and nighttime lights. Data on nighttime lights came from the National Oceanographic and Atmospheric Administration's Nighttime Lights Time Series, which are annual cloud-free composites made from archived DMSP-OLS data, available at a resolution of 30-arc-seconds [30]. These data are available from 1992-2013, as average visible lights, stable lights, and a normalized version of average lights. We used average visible lights, and for model fitting and prediction for 2014-2020 we used data from 2013.

In addition to model selection via leave one out cross-validation as detailed in S1 File, for external validation we also performed spatial regression to check for association between our final wealth index and the proportion of the population earning under $2 per day in 2010. These latter estimates are produced by WorldPop using Bayesian model-based geostatistics and household survey data from the LSMS program, and are available at a resolution of 0.00833° [31]. We found that a one unit increase in proportion earning less than $2/day (i.e., going from 0 to 1) was associated with a 0.84 lower wealth score as predicted by our final Malawi model and a 0.87 lower wealth score as predicted by our final Uganda model, indicating good agreement between our wealth maps and the WorldPop poverty maps. WorldPop does not produce these estimates for DRC.

South Sudan In South Sudan we produced direct estimates of county-level wealth and design-based variance using the `svydesign()` and `svyby()` functions in the `survey` package and household weights provided in the IPUMS extract. In contrast with our livestock mapping methodology detailed in S1 File we did not perform smoothing as our primary goal was not to estimate wealth, thus stabilizing variance of this estimate was not critical.

BIBLIOGRAPHY

[1] Malawi. Demographic and Health Survey 2000. National Statistical Office. Zomba, Malawi. ORC Macro. Calverton, Maryland, USA; August 2001. Available from: https://dhsprogram.com/pubs/pdf/FR123/FR123.pdf.

[2] World Health Organization. World Health Survey Results- Report of Malawi; 2003. Available from: https://www.who.int/healthinfo/survey/whsmwi-malawi.pdf.

[3] Malawi Second Integrated Household Survey (IHS-2) 2004-2005. Basic Information Document. National Statistical Office. Zomba, Malawi; October 2005. Available from: https://microdata.worldbank.org/index.php/catalog/2307/download/35042.

[4] Malawi. Demographic and Health Survey 2004-2005. National Statistical Office. Zomba, Malawi. ORC Macro. Calverton, Maryland, USA; December 2005. Available from: https://dhsprogram.com/pubs/pdf/FR175/FR-175-MW04.pdf.

[5] Technology Adoption and Risk Initiative Household Baseline Survey. The World Bank Microdata Library; 2006. Available from: https://microdata.worldbank.org/index.php/catalog/1541/study-description.

[6] Malawi. Demographic and Health Survey 2010. National Statistical Office. Zomba, Malawi. ORC Macro. Calverton, Maryland, USA; September 2011. Available from: https://dhsprogram.com/pubs/pdf/FR247/FR247.pdf.

[7] Malawi Third Integrated Household Survey (IHS-3) 2010-2011. Basic Information Document. National Statistical Office. Zomba, Malawi; March 2012. Available from: https://microdata.worldbank.org/index.php/catalog/1003/download/40803.

[8] Malawi Integrated Household Panel Survey 2013. Basic Information Document. National Statistical Office. Zomba, Malawi; September 2014. Available from: https://microdata.worldbank.org/index.php/catalog/2939/download/48123.

[9] Malawi Integrated Household Panel Survey 2016. Basic Information Document. National Statistical Office, Zomba, Malawi; November 2017. Available from: https://microdata.worldbank.org/index.php/catalog/2939/download/48116.

[10] Malawi. Malaria Indicator Survey 2012. National Malaria Control Programme, Ministry of Health, Lilongwe, Malawi. MEASURE DHS, ICF International, Rockville, Maryland, USA; November 2012. Available from: https://dhsprogram.com/pubs/pdf/MIS13/MIS13.pdf.

[11] Multiple Indicator Cluster Survey (MICS); UNICEF. Available from: https://mics.unicef.org/.

[12] Malawi. Malaria Indicator Survey 2014. National Malaria Control Programme, Ministry of Health, Lilongwe, Malawi. The DHS Program, ICF International, Rockville, Maryland, USA; February 2015. Available from: https://dhsprogram.com/pubs/pdf/MIS18/MIS18.pdf.

[13] Malawi. Demographic and Health Survey 2015-2016. National Statistical Office. Zomba, Malawi. The DHS Program, ICF International, Rockville, Maryland, USA; February 2017. Available from: https://dhsprogram.com/pubs/pdf/FR319/FR319.pdf.

[14] Malawi Fourth Integrated Household Survey (IHS-4) 2016-2017. Basic Information Document. National Statistical Office. Zomba, Malawi; November 2017. Available from: https://microdata.worldbank.org/index.php/catalog/2936/download/47116.

[15] Malawi. Malaria Indicator Survey 2017. National Malaria Control Programme, Ministry of Health, Lilongwe, Malawi. The DHS Program, ICF International, Rockville, Maryland, USA; January 2018. Available from: https://dhsprogram.com/pubs/pdf/MIS28/MIS28.pdf.

[16] The fifth population census in Sudan: a census with a full coverage and high accuracy; 2008. Available from: https://catalog.ihsn.org/index.php/catalog/4216/download/55707.

[17] République Démocratique du Congo. Enquéte Démographique et de Santé (EDS-RDC) 2007. Ministère du Plan and Ministère de la Santé, and Macro International Inc. Kinshasa, DRC and Calverton, Maryland; August 2008. Available from: https://dhsprogram.com/pubs/pdf/FR208/FR208.pdf.

[18] République Démocratique du Congo. Deuxiéme Enquéte Démographique et de Santé (EDS-RDC) 2013. Ministère du Plan et Suivi de la Mise en oeuvre de la Révolution de la Modernité, Ministère de la Santé Publique, and Measure DHS International. Rockville, Maryland; September 2014. Available from: https://dhsprogram.com/pubs/pdf/FR300/FR300.pdf.

[19] Uganda. Demographic and Health Survey 2001. Uganda Bureau of Statistics. Kampala, Uganda. ORC Macro. Calverton, Maryland, USA; December 2001. Available from: https://dhsprogram.com/pubs/pdf/FR128/FR128.pdf.

[20] Uganda. Demographic and Health Survey 2006. Uganda Bureau of Statistics. Kampala, Uganda. ORC Macro. Calverton, Maryland, USA; August 2007. Available from: https://dhsprogram.com/pubs/pdf/FR194/FR194.pdf.

[21] Uganda. Malaria Indicator Survey 2009. Uganda Bureau of Statistics, Uganda Malaria Surveillance Project Molecular Laboratory, and National Malaria Control Programme, Kampala, Uganda. ICF Macro, Calverton,, Maryland, USA; August 2010. Available from: https://dhsprogram.com/pubs/pdf/MIS6/MIS6.pdf.

[22] The Uganda National Panel Survey (UNPS) 2009/10. Basic Iinformation Document. Uganda Bureau of Statistics.; April 2007. Available from: https://microdata.worldbank.org/index.php/catalog/1001/related-materials.

[23] Uganda. AIDS Indicator Survey 2011. Ministry of Health, Kampala, Uganda, ICF international, Calverton, Maryland, USA, Centers for Disease Control and Prevention, Entebbe, Uganda, US Agency for International Development, Kampala, Uganda, WHO Uganda, Kampala, Uganda; August 2012. Available from: https://dhsprogram.com/publications/publication-AIS10-AIS-Final-Reports.cfm.

[24] Uganda. Demographic and Health Survey 2011. Uganda Bureau of Statistics. Kampala, Uganda. ORC Macro. Calverton, Maryland, USA; August 2012. Available from: https://dhsprogram.com/pubs/pdf/FR264/FR264.pdf.

[25] Uganda. Malaria Indicator Survey 2014-15. Uganda Bureau of Statistics, Uganda Malaria Surveillance Project Molecular Laboratory, and National Malaria Control Programme, Kampala, Uganda. ICF Macro, Calverton,, Maryland, USA; October 2015. Available from: https://dhsprogram.com/pubs/pdf/MIS21/MIS21.pdf.

[26] Uganda. Demographic and Health Survey 2016. Uganda Bureau of Statistics. Kampala, Uganda. ORC Macro. Calverton, Maryland, USA; January 2018. Available from: https://dhsprogram.com/pubs/pdf/FR333/FR333.pdf.

[27] Uganda. Malaria Indicator Survey 2018-19. Uganda Bureau of Statistics, Uganda Malaria Surveillance Project Molecular Laboratory, and National Malaria Control Programme, Kampala, Uganda. ICF Macro, Calverton,, Maryland, USA; March 2020. Available from: https://dhsprogram.com/pubs/pdf/MIS34/MIS34.pdf.

[28] Rutsein SO, Kiersten J. The DHS Wealth Index. DHS Comparative Reports No. 6; 2004. Calverton, Maryland, USA: ORC Macro. Available from: https://www.dhsprogram.com/topics/wealth-index/Wealth-Index-Construction.cfm.

[29] Revelle W. psych: Procedures for Psychological, Psychometric, and Personality Research; 2019. Available from: https://CRAN.R-project.org/package=psych.

[30] Version 4 DMSP-OLS Nighttime Lights Time Series. National Oceanographic and Atmospheric Administration;. Available from: https://ngdc.noaa.gov/eog/dmsp/downloadV4composites.html.

[31] Tatem AJ, Campiz N, Gething PW, Snow RW, Linard C. The effects of spatial population dataset choice on estimates of population at risk of disease. Population Health Metrics. 2011;9(4). doi:http://www.pophealthmetrics.com/content/9/1/4.
