# Supplementary material for: The effect of livestock density on Trypanosoma brucei gambiense and T. b. rhodesiense: A causal inference-based approach
Source: PLoS Negl Trop Dis. 2022 Aug 29;16(8):e0010155. doi: 10.1371/journal.pntd.0010155 (PMC9462671; doi:10.1371/journal.pntd.0010155)
Supplement: S2 Appendix — (PDF) [file pntd.0010155.s002.pdf]

S2 Appendix: Motivation and implementation of the parametric g-formula

Identifiability criteria

By notational convention, let L be a vector of measured confounders, Y be outcome, A be exposure, and T be time, and let overbars denote history through time $T = t$. The idenfiability criteria are exchangeability, positivity, and the stable unit treatment value assumption (SUTVA).

As exchangeability, also known as no exposure-outcome confounding, is rarely satisfied marginally, the weaker assumption of exchangeability conditional on the past and on L is typically adopted. Using counterfactual notation, this assumption is expressed as $Y^{\overline{a}} \perp A_t | \overline{A}_{t-1}, \overline{L}_t$.

Positivity, which refers to a positive probability of being assigned to each of the treatment levels (that is, no structural zeroes) is expressed as $Pr(A = a|L = l) > 0$ for $Pr(L = l) \neq 0$.

SUTVA has two components: consistency and no interference. Under consistency, each unit of observation (here, a cluster-year) has one potential outcome for a given treatment level $A = a$, and therefore a cluster's observed outcome equals their counterfactual outcome under their observed exposure: if $A_i = a$, then $Y_i^a = y_i$ for all clusters. Under no interference, each cluster's potential outcome is independent of all other clusters' potential outcomes. In our study we instead assume partial interference, detailed in the main text.

Implementation

Let $\overline{A} = \overline{a}^*$ be the deterministic regime under which A is set to a^* at all intervals $t \in \{1, ..., T\}$. The g-formula is given as [1]:

$$E[Y_t^{\overline{a}}] = \int_{\bar{l}} E[Y_t | \overline{A} = \overline{a}^*, \overline{L} = \bar{l}] \prod_{t=0}^{T} f\{l_t | \overline{A}_t = \overline{a}_t^*, \overline{L}_{t-1} = \bar{l}_{t-1}\}$$

$E[Y_t^{\overline{a}}]$ is estimated by simulating the joint distribution of $\overline{L}$ and Y that would have been observed had all units received exposure $\overline{A} = \overline{a}$ (i.e., model-based standardization). First,

we defined our causal estimand $E[Y^a/Y^{a^*}]$, where a^* = empirical mean livestock density, and $a = 1.5 \times$ empirical mean livestock density.

Next, we examined our directed acyclic graph, contained in the main text, to identify the causal sequence of our time-varying variables, in order to write down the model for each time-varying confounder (wealth, NDVI, and LST) and the outcome (HAT cases). We handled partial interference by adjusting for mean livestock density in the interference set in the outcome (HAT) models.

Finally, we tied each of these models together by invoking the law of total probability, allowing us to obtain a marginal probability by averaging over conditional probabilities. Using the notation given above, a simplified version of the outcome model is given as:

$$E[Y] = \sum_A \sum_L E[Y|A, L]P(A|L)P(L) \tag{2.1}$$

As we will implement the g-formula by setting $A = a$, we can remove $P(A|L)$ from Eq 2.1. Under the identifiability criteria, we can thus identify the counterfactual parameter $E[Y^a]$:

$$E[Y^a] = \sum_L E[Y|A = a, L]P(L) \tag{2.2}$$

As the first term in Eq 2.2 corresponds to our outcome regression model, we need to marginalize over $P(L)$ to get $E[Y^a]$. We do this by modeling the joint distribution of all confounders (time-varying and fixed) by taking 10,000 Monte Carlo samples from the observed data and using these samples for prediction (detailed below), ensuring all clusters which reported cases were retained. We used non-parametric bootstrapping (100 bootstrapped samples) to obtain confidence intervals.

We implemented all models using the `glm()` function in R. We did not use spatial models due to computational challenges and concerns regarding spatial confounding. We fit Gaussian models for wealth, NDVI, and LST, and Poisson models for HAT cases. Our procedure was as follows, iterating over each t with t_0 defined as 2001 (2000 was not modeled due to the need to implement lags):

1. For t_0, fit all models except the outcome model (HAT cases): wealth, NDVI, and LST. Outcome model not fit due to the need to lag livestock by two time points (years)

2. Set livestock density to $A = a$, and for each model in turn use the `predict()` function to predict the variable in question under $A = a$ on the Monte Carlo sample
3. Repeat for all times $t_1, ..., t_T$, adding in the outcome model for $t > 0$.
4. Iterate steps 1-3 over each bootstrapped sample

The result of this procedure is a vector of counterfactual outcomes for each simulation s, with $Y^{\overline{a}}_{t(s)i}$ defined as the 1-year cumulative incidence of HAT in cluster i, year t, simulation s, and with exposure set to $\overline{A} = \overline{a}$.

$$\mathbf{Y}^{\overline{a}}_{(s)i} = \left\{ Y^{\overline{a}}_{1(s)i}, Y^{\overline{a}}_{2(s)i}, ..., Y^{\overline{a}}_{T(s)i} \right\}$$

As noted above, we take the ratio of the counterfactual outcomes under $\overline{A} = \overline{a}$ versus under $\overline{A} = \overline{a}^*$ as the target of inference. Across clusters, time and simulations, we implement this as follows, separately for each country (and for gHAT and rHAT in Uganda):

$$E_{(s)}\left[E_t\left[E_i[Y^{\overline{a}}_{t(s)i}] \, / \, E_i[Y^{\overline{a}^*}_{t(s)i}] \right] \right]$$

with uncertainty bounds estimated by taking the 2.5% and 97.5% quantiles across simulations.

BIBLIOGRAPHY

[1] Robins JM, Hernan MA. 23. In: Estimation of the causal effects of time-varying exposures. Boca Raton: CRC Press; 2009.
