# Supplementary material for: The effect of livestock density on Trypanosoma brucei gambiense and T. b. rhodesiense: A causal inference-based approach
Source: PLoS Negl Trop Dis. 2022 Aug 29;16(8):e0010155. doi: 10.1371/journal.pntd.0010155 (PMC9462671; doi:10.1371/journal.pntd.0010155)
Supplement: S3 Appendix — (PDF) [file pntd.0010155.s003.pdf]

S3 Appendix: Implementation of the measurement error model in South Sudan

The measurement error (MEC) model is defined as:

$$w_c = x_c + u_c, c = 1, ..., n$$

where c indexes county, u_c are the measurement error terms distributed as $u_c \sim N(0, \sigma_u^2)$, and x_c and u_c are independent. Since the u terms have mean 0, $E(W|X = x) = x$, that is, W is unbiased for a given unobserved x. Failing to account for measurement error will result in biased effect estimates and inappropriate standard errors [1].

Following the heteroscedastic errors-in-variables approach detailed in Wang et al. (2018), we defined our hierarchical model as follows:

$$Y_c|\lambda = exp\Big(\beta_0 + \beta_1\mu_c + \beta_2\eta_c + \boldsymbol{\alpha}\mathbf{z}(\mathbf{k}_c) + S_c + \epsilon_c + log(P_c)\Big)$$

$$\mu_c = x_{lc} + u_{lc}, \eta_c = x_{wc} + u_{wc}$$

$$x_{lc} = \lambda_l + \zeta_{lc}, x_{wc} = \lambda_w + \zeta_{wc}$$

$$\epsilon_c|\sigma_\epsilon^2 \sim_{iid} N(0, \sigma_\epsilon^2)$$

$$u_{lc} \sim N(0, d_c\sigma_{ul}^2), u_{wc} \sim N(0, d_c\sigma_{uw}^2)$$

$$\zeta_{lc} \sim N(0, \sigma_{xl}^2), \zeta_{wc} \sim N(0, \sigma_{xw}^2)$$

where

- Y_c is the number of cases in county c
- μ_c is estimated livestock (cattle or pig) density in county c
- η_c is estimated wealth score in county c
- $\boldsymbol{\alpha}$ is a vector of coefficients
- $\mathbf{z}(\mathbf{k}_c)$ is a vector of confounders measured without error

- P_c is the offset, given as population in county c
- ϵ_c are county-level iid (unstructured) random effects with variance σ_ϵ^2
- S_c are county-level structured random effects which follow the ICAR model with marginal variance σ_s^2
- $ne(c)$ denotes neighbors (shared boundary) of county c
- m_c is the number of neighbors of county c
- x_{lc} is the true livestock density in county c
- u_{lc} is the measurement error for livestock density in county c
- x_{wc} is the true wealth score in county c
- u_{wc} is the measurement error for wealth score in county c
- λ_l is the mean of the true livestock density
- λ_w is the mean of the true wealth score
- ζ_{lc} is the residual for livestock density in county c
- ζ_{wc} is the residual for wealth score in county c
- d_c is a weight which allows for heteroscedasticity in the error structure

For both livestock density and wealth score, we assume $x_c \sim N(\theta, \sigma_x^2)$, where θ is the mean of x_c and σ_x^2 is the variance.

There are therefore four parameters needed for each of the two resulting measurement error models: β_1 (β_2 for wealth score), $\log(1 / \sigma_u^2)$, λ, $\log(1 / \sigma_x^2)$, as well as the scale factor d_c. For livestock density, we specified the priors and starting values for these parameters as follows for livestock, substituting μ for η and β_1 for β_2 for wealth:

- $\log(1 / \sigma_u^2) \sim \text{logGamma}(10, 10)$, starting value $\log\big(1 / var(\sigma_\mu)\big)$
- $\beta_1 \sim \text{Normal}(\hat{\beta}_1, 1 / \hat{\sigma}^2_{\hat{\beta}_1})$.
- λ fixed at mean $E[\mu]$, with a Gaussian prior
- $\log(1 / \sigma_x^2) \sim \text{logGamma}(10, 10)$, starting value $\log(1 / var(\mu))$
- $d_c \sim \text{Unif}(0.5, 1.5)$

where $var(\sigma_\mu)$ is the empirical variance of the posterior standard deviation for livestock density, $\hat{\beta}_1$ is the posterior mean of the coefficient for livestock density from the naive model, $\hat{\sigma}_{\hat{\beta}_1}$ is the posterior standard deviation of the same coefficient from the naive model, and $var(\mu)$ is the empirical variance of the posterior median for livestock density. The logGamma prior specification is equivalent to that used in Wang et al. [1]. The MEC model for wealth was specified equivalently, and in our final models both livestock density and wealth were included in this form.

BIBLIOGRAPHY

[1] Wang X, Yue YR, Faraway JJ. 10. In: Errors-in-Variables Regression. Boca Raton, FL: Chapman & Hall/CRC. Computer Science and Data Analysis Series; 2018.
